# The histone modifier KAT2A presents a selective target in a subset of well-differentiated microsatellite-stable colorectal cancers

**DOI:** 10.1038/s41418-025-01479-7

**Published:** 2025-03-27

**Authors:** Vida Kufrin, Annika Seiler, Silke Brilloff, Helen Rothfuß, Sandra Küchler, Silvia Schäfer, Elahe Rahimian, Jonas Baumgarten, Li Ding, Frank Buchholz, Claudia R. Ball, Martin Bornhäuser, Hanno Glimm, Marius Bill, Alexander A. Wurm

**Affiliations:** 1 Mildred Scheel Early Career Center, National Center for Tumor Diseases (NCT/UCC) Dresden, Faculty of Medicine and University Hospital Carl Gustav Carus, TUD Dresden University of Technology, Dresden, Germany; 2 Department of Translational Medical Oncology, National Center for Tumor Diseases (NCT/UCC) Dresden, a partnership between DKFZ, Faculty of Medicine of the TUD Dresden University of Technology, University Hospital Carl Gustav Carus Dresden, and Helmholtz-Zentrum Dresden - Rossendorf (HZDR), Dresden, Germany; 3 Medical Systems Biology, UCC, Medical Faculty Carl Gustav Carus, TUD Dresden University of Technology, Dresden, Germany; 4 Translational Medical Oncology, Faculty of Medicine and University Hospital Carl Gustav Carus, TUD Dresden University of Technology, Dresden, Germany; 5 German Cancer Consortium (DKTK), Dresden, Germany; 6 TUD Dresden University of Technology, Faculty of Biology, Dresden, Germany; 7 Department of Internal Medicine I, University Hospital Carl Gustav Carus, TUD Dresden University of Technology, Dresden, Germany; 8 Translational Functional Cancer Genomics, National Center for Tumor Diseases (NCT) Heidelberg and German Cancer Research Center (DKFZ), Heidelberg, Germany

**Keywords:** Tumour biomarkers, Translational research

## Abstract

Lysine acetyltransferase 2 A (KAT2A) plays a pivotal role in epigenetic gene regulation across various types of cancer. In colorectal cancer (CRC), increased *KAT2A* expression is associated with a more aggressive phenotype. Our study aims to elucidate the molecular underpinnings of *KAT2A* dependency in CRC and assess the consequences of *KAT2A* depletion. We conducted a comprehensive analysis by integrating CRISPR-Cas9 screening data with genomics, transcriptomics, and global acetylation patterns in CRC cell lines to pinpoint molecular markers indicative of *KAT2A* dependency. Additionally, we characterized the phenotypic effect of a CRISPR-interference-mediated *KAT2A* knockdown in CRC cell lines and patient-derived 3D spheroid cultures. Moreover, we assessed the effect of *KAT2A* depletion within a patient-derived xenograft mouse model in vivo. Our findings reveal that *KAT2A* dependency is closely associated with microsatellite stability, lower mutational burden, and increased molecular differentiation signatures in CRC, independent of the *KAT2A* expression levels. *KAT2A-*dependent CRC cells display higher gene expression levels and enriched H3K27ac marks at gene loci linked to enterocytic differentiation. Furthermore, loss of *KAT2A* leads to decreased cell growth and viability in vitro and in vivo, downregulation of proliferation- and stem cell-associated genes, and induction of differentiation markers. Altogether, our data show that a specific subset of CRCs with a more differentiated phenotype relies on KAT2A. For these CRC cases, KAT2A might represent a promising novel therapeutic target.

## Introduction

Colorectal cancer (CRC) continues to be one of the leading causes of cancer-related mortality worldwide, despite considerable advancements in the evolution of treatment modalities [1]. Recent decades have witnessed a pivotal shift in the field of CRC treatment towards precision oncology, offering personalized and more effective cancer care [2]. This approach involves strategic implementation of targeted agents, designed to block the survival and growth of tumor cells by interfering with cancer drivers [3]. Given the persistently poor outcomes for CRC patients, particularly in advanced stage CRCs where promising tumor vulnerabilities are often lacking, there is an urgent need to identify novel therapeutic targets.

One important class of potentially actionable targets encompasses epigenetic modulators, as numerous cancer-associated phenotypes have been linked to alterations in their expression or activity [4, 5]. Within the realm of epigenetic modulators, histone acetyltransferases (HATs) are central in modifying the chromatin structure by adding acetyl groups to lysine residues on histone tails [6]. Particularly, lysine acetyltransferase 2A (KAT2A, also known as GCN5) was the first HAT discovered to provide strong molecular evidence directly linking histone acetylation to gene transcription activation [7, 8]. KAT2A is a member of the Spt-Ada-Gcn5-Acetyltransferase (SAGA) coactivator complex and interacts with SGF29, TADA2B, and TADA3 to facilitate gene expression [9]. It plays a key role in a broad range of cellular processes, including cell proliferation, differentiation, and cell cycle regulation [10]. Importantly, *KAT2A* has been shown to be significantly upregulated in many cancers, including CRC [11], and has been associated with promoting more aggressive phenotypes and progression in different cancer entities [12–14]. Thus, we hypothesize that KAT2A may serve as a promising target with effective therapeutic prospects for various cancer types. Nevertheless, the specific role of KAT2A in CRC remains predominantly unknown and requires further elucidation.

Herein, we demonstrate that a subset of microsatellite-stable (MSS), more-differentiated CRC subtypes depend on KAT2A. Moreover, we identified distinct molecular features of KAT2A dependent CRCs and explored the effects of *KAT2A* depletion on CRC maintenance both in vitro and in vivo. Ultimately, we aimed to characterize KAT2A as a potential novel therapeutic target in CRC.

## Materials and methods

### Cancer Cell Line Encyclopedia (CCLE) and Cancer Dependency Map (DepMap)

Analyses of CRISPR-Cas9-based dependency data, corresponding mRNA expression generated by RNA sequencing, and somatic mutational profiles for 1078 pan-cancer cell lines including 57 CRC cell lines were performed using data from the Cancer Cell Line Encyclopedia (CCLE) and the DepMap Portal (version 22Q4) [15]. Raw data were downloaded and analyzed for each indicated cell line. Summarized data can be found in Supplementary Table S1.

### Gene set enrichment analysis (GSEA)

Differentially expressed genes were calculated by comparing the median expression of log2-modified read counts between KAT2A dependent (dependency score <−0.4, *n* = 12) and independent (dependency score >−0.1, *n* = 18) CRC cell lines for all annotated genes. These values were then plotted as log2 fold changes against the logarithmically converted p-values obtained from a two-sided unpaired *t*-test. Gene set enrichment analysis (GSEA) was performed using GSEA software (http://www.broadinstitute.org/gsea/) according to the publisher’s instructions [16]. Indicated enterocyte signatures were part of the gene ontology gene sets (C8) for cell type signature gene sets. Summarized data can be found in Supplementary Table S2.

### Cell culture

HT29 CRC cell line was obtained from DSMZ and continuously tested for mycoplasma and cross contaminations by Multiplex Cell Authentication (Multiplexion, Heidelberg, Germany). They were cultured in McCoy’s 5A medium supplemented with 10% FBS and 1% penicillin/streptomycin. HEK293TN cells were obtained from System Biosciences and cultured in DMEM supplemented with 10% FBS. Patient-derived 3D spheroids (CRC1, CRC2, CRC3) were cultured as previously described [3]. All samples were obtained from the University Hospital Heidelberg. All patients provided written informed consent in accordance with the Declaration of Helsinki. The study protocols used for patient sample collection were approved by Ethical Committee of each University. For generation of the spheroid cultures, primary tumor samples were dissociated as previously published [3]. Singularized cells were cultured in ultra-low attachment flasks (Corning, NY, USA) in serum-free culture medium (Advanced DMEM/F-12 supplemented with 0.6% glucose, 2 mM l-glutamine (ThermoFisher Scientific, Waltham, MA, USA), 5 mM HEPES, 4 µg/mL heparin (Sigma-Aldrich, St. Louis, MO, USA), 4 mg/mL bovine serum albumin (PAN-Biotech, Aidenbach, Germany)). Human recombinant EGF (20 ng/mL) and human recombinant FGF (10 ng/mL) (R&D Systems, Minneapolis, MN, USA) were added twice per week.

### CRISPR-interference-based *KAT2A* knockdown

dCas9-KRAB-expressing CRC cells were generated by lentiviral transduction with pLV hUbC-dCas9 KRAB-T2A-GFP, a kind gift from Charles Gersbach (Addgene #67620) [17]. Two sgRNAs targeting *KAT2A*, designed using CRISPick sgRNA designer from the Broad Institute [18, 19], and one non-targeting control sgRNA (NTC) were cloned into an BFP-tagged pCRISPRia-v2 vector, a gift from Jonathan Weissman (Addgene #84832) [20]. sgRNAs were lentivirally transduced into dCas9-KRAB-expressing CRC cells. Two days after transduction, the cells were separated into two fractions: one part was selected with puromycin for knockdown assessment, whereas the rest of the cells were cultured for competition assay. The following sgRNA sequences were used: sgRNA1: TCCCAGCCCTAGGGCCGCAT; sgRNA2: GCGCCGCGCTCCCAGCCCTA; NTC sgRNA: GCGATCTAATCGGAACTGTG.

### Flow cytometry and competition assay

For flow cytometry analysis, dCas9-KRAB-expressing cells were transduced with the respective sgRNAs. Four days post transduction, cells were washed with PBS and measured with BD LSR Fortessa cell analyzer to determine transduction efficiency, which also served as day 0 baseline. Cells were reanalyzed every 4 days for growth assessment.

### RNA isolation, cDNA synthesis and quantitative real-time PCR (qPCR)

RNA was isolated using RNAeasy mini kit (Qiagen, Hilden, Germany). Reverse transcription (RT) was performed with the RevertAid First Strand cDNA Synthesis Kit (Life Technologies, Carlsbad, CA, USA) according to the manufacturer’s instructions. For qPCR, SsoAdvanced# Universal SYBR® Green Super Mastermix (Bio-Rad, Hercules, CA, USA) was used. The following primers were included: *KAT2A*-for 5′-GTCTTCTCGGCTTGCAAGGC-3′; *KAT2A*-rev 5′-TGCTTGGTGTCTGTGTCCTC-3′; *ACTB*-for 5′-CATTGCTGACAGGATGCAGAAGG-3′; *ACTB*-rev 5′-TGCTGGAAGGTGGACAGTGAGG-3′.

### Protein isolation and western blot

Western blots were performed as previously described [21]. Briefly, proteins were isolated by cell lysis in lysis buffer (150 mM NaCl, 50 mM Tris-HCL, 1% NP-40, 0.5% Sodium deoxycholade and 0.1% SDS) with protease inhibitor cocktail and phosphatase inhibitor cocktail. Protein concentration was measured using Bio-Rad Protein Assay (Bio-Rad). Denatured proteins were run on a Tris-Glycine SDS-Polyacrylamide gel according to the Bio-Rad protocol and transferred to PVDF membranes. Primary antibody incubation was performed at 4 °C overnight. Secondary antibody was incubated for 1 h at room temperature. The following antibodies were used: Anti-GCN5L2 (C26A10) Rabbit mAb (#3305, CST, Danvers, MA, USA), anti-GAPDH (D16H11) XP Rabbit mAb (#5174, CST) and anti-Cas9 (S. pyogenes) (7A9-3A3) Mouse mAb (#14697, CST). Rabbit Anti-Mouse-HRP (ab6728, Abcam) and Goat Anti-Rabbit-HRP (ab6721, Abcam) served as secondary antibodies. Uncropped blots are shown in Supplementary Material File.

### Doxycycline-inducible *KAT2A* knockdown after 3D spheroid formation

Inducible dCas9-KRAB-expressing CRC3 cells were generated by lentiviral transduction with pHAGE TRE dCas9-KRAB, a kind gift from Rene Maehr & Scot Wolfe (Addgene #50917) [22]. Subsequently, G418-selected inducible CRC3-dCas9-KRAB cells were transduced with sgRNA1 targeting *KAT2A*, or NTC as control, and selected with puromycin. To knockdown *KAT2A* after spheroid formation, cells from both the sgKAT2A and sgNTC group were seeded in 384 wells at nearly single cell level into individual wells. After 48 h and after cells formed visible 3D spheroid structures, doxycycline (5 µg/ml) was added to induce *KAT2A* knockdown. Spheroid growth was monitored by ImageXpress Confocal HT.ai system (Molecular Devices, San Jose, CA, USA) at four and seven days after initial doxycycline administration. Only wells with visible spheroids were taken into consideration. Spheroid sizes for each individual well was determined using ImageJ.

### Library preparation for RNA sequencing and data analysis

RNA sequencing libraries were prepared using the Stranded mRNA Prep, Ligation Kit (Illumina, San Diego, CA, USA) according to the manufacturer’s instructions. Briefly, mRNA was purified from 1 µg total RNA using oligo(dT) beads, poly(A) + RNA was fragmented to 150 bp and converted into cDNA, and cDNA fragments were end-repaired, adenylated on the 3’ end, adapter-ligated, and amplified with 12 cycles of PCR. The final libraries were validated using a Qubit 2.0 Fluorometer (Life Technologies) and a Bioanalyzer 2100 system. All barcoded libraries were pooled and sequenced 2x75bp paired-end on an Illumina NextSeq550 platform to obtain a minimum of 10 Mio reads per sample.

Quality control assessment of raw reads was performed using FastQC, followed by Trimmomatic trimming to remove low-quality sequences. The trimmed reads were then aligned to the reference genome using STAR version 2.7.9a. To facilitate the mapping process, a genome reference index was constructed using either GRCh37.fa (hg19) or GRCh38.108.gtf (hg38) as the reference.

### Drug sensitivity assay, CPTH2 treatment, FACS staining, apoptosis and cell cycle analysis

The response of spheroid models to KAT2A inhibitors, butyrolactone-3 (MB-3; Sigma-Aldrich, St Louis, MO, USA) and cyclopentylidene-[4-(4′-chlorophenyl)thiazol-2-yl]hydrazone (CPTH2; ThermoFisher Scientific), was determined by generation of dose response curves using ATPlite 1step Luminescence Assay System (Parkin Elmer, Waltham, MA, USA) according to manufacturer’s instructions. Briefly, 5 × 10^3^ singularized cells were seeded into a 96-well plate and subsequently treated with MB-3 or CPTH2 in 11 descending concentrations. Following 72-h drug incubation, ATP content as a surrogate for cell viability was determined by measuring luminescence. All following assays were performed using CRC3 model 3 days after treatment with CPTH2 (50 µM) or DMSO as control. Cell cycle analysis was performed by incubating 5 × 10^5^ cells with 5 µg/ml Hoechst-33342 for 60 min at 37 °C and subsequent flow cytometry analysis. For flow cytometry-based EPHB2-staining, 5 × 10^5^ cells were washed with PBS and stained for 20 min at 4 °C with anti-EphB2 APC conjugated antibody (BD Biosciences, Heidelberg, Germany). Apoptosis assay was performed using Annexin V-FITC Kit (Miltenyi Biotec, Bergisch Gladbach, Germany) according to the manufacturer’s instructions.

### In vivo sgRNA xenografts

Animal experiments were approved according to institutional guidelines and German animal welfare regulations. Mice were bred within the NCT/UCC Dresden animal facility at the University Hospital Dresden in standard individually ventilated cages according to the current hygiene and animal welfare guidelines. For xenograft development, CRC3-dCas9-KRAB spheroids were lentivirally transduced with KAT2A sgRNA1 or NTC sgRNA. After puromycin selection, 5 × 10^5^ cells were mixed 1:1 with Matrigel in a total volume of 150 μL and subcutaneously injected in flanks of NOD/SCID/gamma (NSG) mice (The Jackson Laboratory) (*n* = 5 per group). After tumors started to form, tumor volume was measured every 4 days with a vernier caliper. After tumors reached the maximum size of 13 mm in diameter, mice were sacrificed, and tumors were excised, photographed, weighted, and fixed in 4% PFA for Hematoxylin and Eosin (HE) staining and immunohistochemical analysis with antibodies against KI67, CDX2, KRT20 and VIL1.

### Analysis of external ChIP-Seq datasets

Raw BED data from published ChIP-Seq datasets [23, 24] were downloaded from Gene Expression Omnibus (GEO) with the Accession Numbers GSE73319 and GSE83968, and further processed using the open access EaSeq software [25] according to the published guidelines.

### Analysis of external datasets

We analyzed mRNA expression from 357 patients with colon adenocarcinoma (TCGA-COAD) from the Cancer Genome Atlas (TCGA) database [26] and 110 patients diagnosed with CRC generated by Clinical Proteomic Tumor Analysis Consortium [27]. Raw data were downloaded using cBioportal online tool (https://www.cbioportal.org/) and further processed according to the median expression of the studied genes.

### Statistical analysis

We used Wilcoxon rank sum test (also known as Mann–Whitney *U* test) or two-sided unpaired *t*-test to determine the statistical significance of experimental results. The results were represented as the mean ± SD from at least three independent experiments unless elsewise stated. To compare Kaplan–Meier survival curves, we applied Log-rank (Mantel–Cox) test. A *p* value of 0.05 or less was considered significant. All data were plotted using GraphPad Prism version 8.1.2.

## Results

### *KAT2A* dependency in CRC cell lines exhibits no discernible correlation with *KAT2A* expression but is intricately associated with a reduced mutational burden

**Fig. 1:**
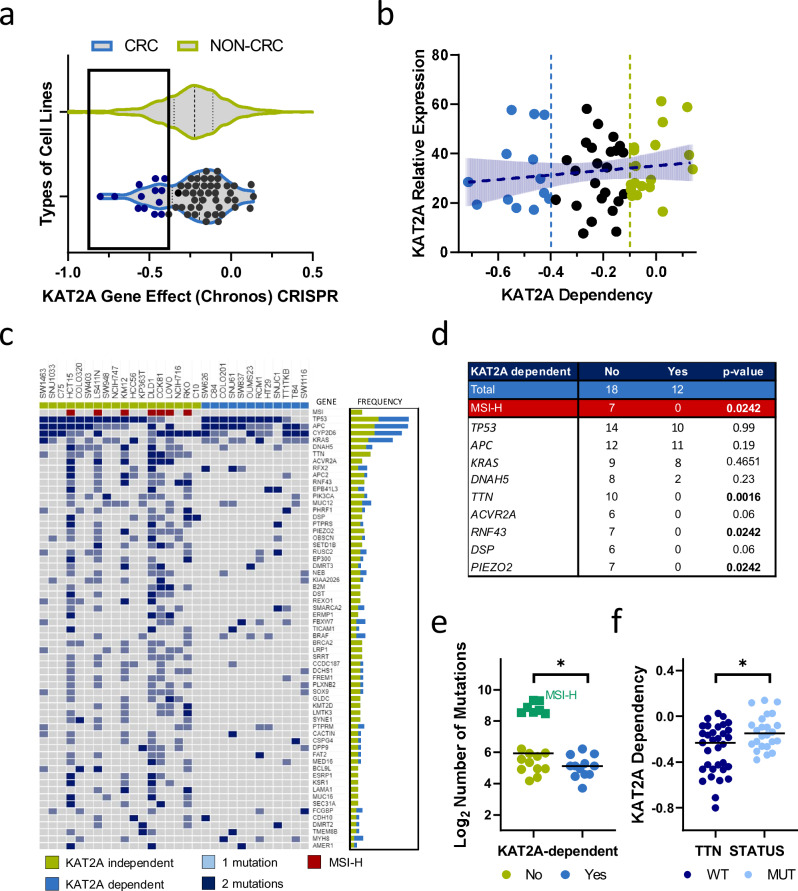
*KAT2A* dependency in CRC cell lines does not correlate with *KAT2A* expression but is linked to a lower mutational burden. **a** Violin plot of *KAT2A* dependency scores from the DepMap data set. In blue are all colorectal cancer cell lines (*n* = 57). In green are all other included cancer cell lines (*n* = 1078). The dashed line indicates the median for each, and the quartiles are shown as dotted lines. The *KAT2A* dependent CRC lines are indicated in dark blue. **b** Correlation of *KAT2A* expression (*y*-axis) and *KAT2A* dependency scores (x-axis) in all CRC cell lines. They were stratified into *KAT2A* dependent (blue), intermediate (black), and *KAT2A* independent (green) cell lines. **c** Oncoplot illustrating mutational pattern of *KAT2A* independent (green, *n* = 18) and *KAT2A* dependent (blue, *n* = 12) CRC cell lines. Light blue indicates 1 mutation, dark blue >1 mutations per gene (**d**) Table overview of the distribution of most frequent gene mutations and microsatellite-instability (MSI) status between the two groups. **e** Total number of mutations between the two groups, and (**f**) *KAT2A* dependency between *TTN*-wild-type (WT, *n* = 33) and *TTN*-mutant (*n* = 24) CRCs. *p < 0.05 two-tailed Mann–Whitney *U* test.

To explore KAT2A as a potential target in CRC, we took advantage of the DepMap’s public genome-scale CRISPR-Cas9 22Q4 (Chronos) essentiality screen dataset [15]. In this dataset, the main measure is the dependency score, which indicates the relative effect of target gene perturbation on cell proliferation, scaled per cell line. A lower score denotes a higher dependency rate, and a score lower than −0.5 for individual genes is commonly deemed as entirely essential [28]. By comparing the dependency scores of *KAT2A* across a cohort of CRC cell lines with those of all other cell lines contained in the dataset, we identified a subset of CRC cell lines showing greater relative dependency (dependency score < −0.4). This finding suggests that *KAT2A* may represent a specific vulnerability for a subset of CRC cell lines (Fig. 1a and Supplementary Fig. S1a).

To elucidate the mechanism underlying this difference, we categorized CRC cell lines into three distinct groups according to their respective dependency score; *KAT2A* dependent (marked in blue, dependency score < −0.4), *KAT2A* intermediate (marked in black, dependency score between −0.4 and −0.1), and *KAT2A* independent (marked in green, dependency score > −0.1). As an initial step, we sought to determine whether differential dependency could be attributed solely to *KAT2A* expression levels. However, no significant correlation could be observed between *KAT2A* dependency and its expression (Fig. 1b and Supplementary Table 1). Thus, we were interested in whether *KAT2A* dependency exhibits an association with common genetic alterations. We compared mutational profiles for most common genetic alterations in CRC between *KAT2A* independent and *KAT2A* dependent groups. Microsatellite instability (MSI) status was also incorporated in the analysis. For driver mutations, specifically *APC*, *TP53*, and *KRAS* mutations [29], we observed no discernible differences between the two groups. However, several genes, such as *TTN*, *RNF43*, and *PIEZO2* were found to be mutated exclusively in *KAT2A*-independent cell lines. Furthermore, among the *KAT2A*-independent CRC cell lines, 7/18 were MSI-high, whereas none of the 12 *KAT2A* dependent CRC cells displayed this characteristic (Fig. 1c and d). Consequently, we also found that *KAT2A*-dependent CRC cell lines generally exhibited a significantly lower mutational burden when compared to *KAT2A*-independent cells, raising the possibility that *KAT2A* may not be a dependency in tumors strongly driven by mutational events, but could instead play a more crucial role in epigenetically driven CRCs (Fig. 1e).

For further validation, we explored the link between *KAT2A* dependency and *TTN* mutational status. In CRC, the mutation load within *TTN* has been established as a predictor of overall mutational burden [30]. Notably, CRC cell models harboring at least one mutation in *TTN* gene displayed a significantly lower dependency score compared to *TTN* wild-type cell models, suggesting that mutational burden is an indicator of *KAT2A* dependency (Fig. 1f). Taken together, these data suggest that *KAT2A* expression alone does not account for *KAT2A* dependency. However, *KAT2A-*dependent CRC cell lines display a lower mutational burden and are exclusively MSS.

### CRC cell lines that rely on *KAT2A* exhibit expression of genes linked to cellular differentiation

**Fig. 2:**
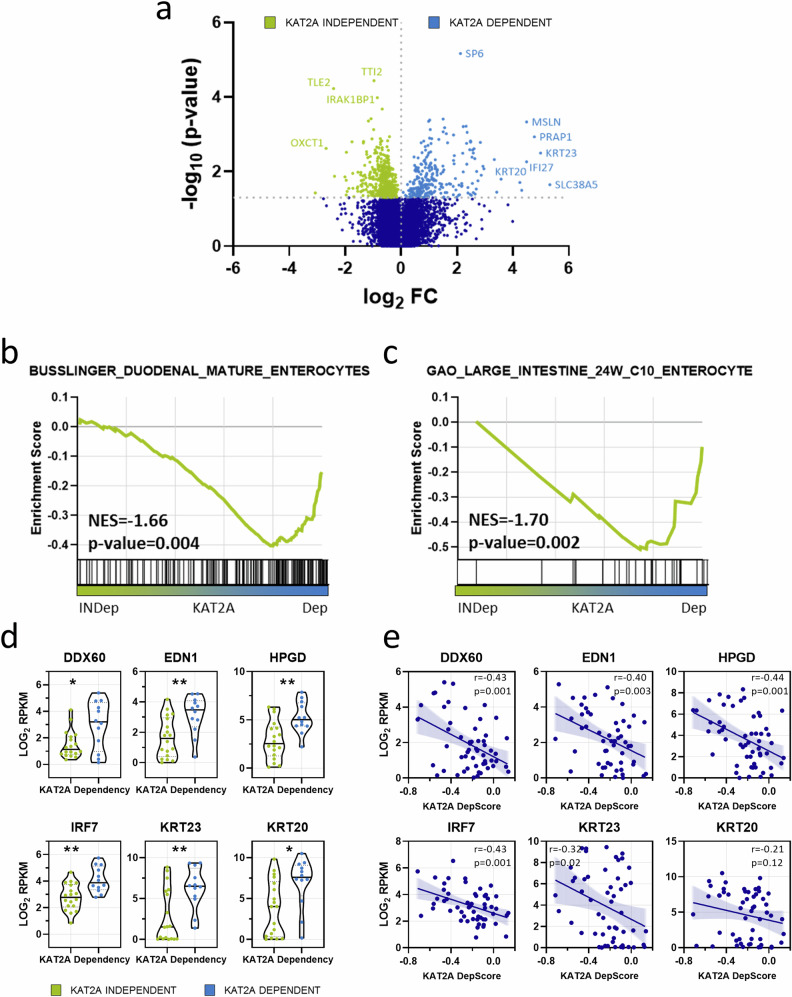
*KAT2A-*dependent CRC cell lines express differentiation-associated genes. **a** Volcano plot analysis of differentially expressed genes between *KAT2A* independent (green) and dependent (blue) CRC cell lines. Strongest hits are highlighted. **b**, **c** Gene set enrichment analysis (GSEA) indicating positive enrichment for gene signatures related to mature enterocytes in *KAT2A*-dependent CRC cell lines. **d** Differential expression of colon differentiation marker genes *DDX60*, *EDN1*, *HPGD*, *IRF7*, *KRT23*, *and KRT20* between the *KAT2A* independent (green) and dependent (blue) groups. *p < 0.05; **p < 0.01, two-tailed Student’s *t*-test. **e** Correlation of relative gene expression of indicated differentiation markers (*y*-axis) and *KAT2A* dependency scores (*x*-axis) in all CRC cell lines. The Pearson correlation coefficients *r* and the *p*-values are shown.

To assess whether active transcriptional programs differ between *KAT2A* independent (*n* = 18) and *KAT2A* dependent (*n* = 12) CRC cell lines, we retrieved the publicly available gene dependency and RNA sequencing-based gene expression dataset from the DepMap portal and conducted correlation analysis. Interestingly, *KAT2A* exhibited a strong co-dependency with other members from the SAGA complex (Supplementary Fig. S1b, c). Moreover, expression analysis revealed a subset of genes that exhibit significantly higher expression levels (*p* < 0.05) within each group, suggesting that there indeed is a global difference in transcriptional profiles of *KAT2A* independent and dependent cell lines (Fig. 2a, Supplementary Fig. S1d, Supplementary Table 1).

Next, we carried out gene set enrichment analysis (GSEA) using the MsigDB C8 Collection cell type signature gene sets as a reference to further identify if observed transcriptional differences between subgroups can be assigned to distinct gene signatures. Remarkably, among the highest-ranked signatures specific to *KAT2A-*dependent cell lines, two individual gene signatures [31, 32] pointing towards mature enterocytes were identified (Fig. 2b and c). Enterocytes are the most abundant cells in the colon, but more importantly, they are recognized to be terminally differentiated [33].

Furthermore, when examining differentially expressed genes at the individual gene expression level between *KAT2A* independent and dependent CRC cells, we observed significantly higher expression of enterocyte-specific marker genes [34] – specifically, *EDN1*, *HPGD*, *IRF7*, *KRT23*, and *DDX60* as well as pan-differentiation marker *KRT20* in the *KAT2A* dependent group (Fig. 2d). Subsequently, we were interested to see if there is a correlation between the expression of these markers and *KAT2A* dependency in all CRC cell lines utilizing the DepMap dataset. As expected, we revealed a consistent correlation between the expression of all aforementioned differentiation markers and *KAT2A* dependency. Notably, the highest correlation was observed for *HPGD* (*r* = −0.44, *p* = 0.001) and *IRF7* (*r* = −0.43, *p* = 0.001). However, for pan-differentiation marker *KRT20*, the correlation was mild (*r* = −0.21), and did not attain statistical significance (*p* = 0.12) (Fig. 2e).

Collectively, these findings imply that *KAT2A*-dependent CRC cells express genes associated with enterocytic differentiation. Moreover, the expression of certain differentiation genes may serve as surrogate marker of *KAT2A* dependency.

### CRISPR interference-mediated knockdown of *KAT2A* reduces tumor growth in MSS patient-derived CRC models

**Fig. 3:**
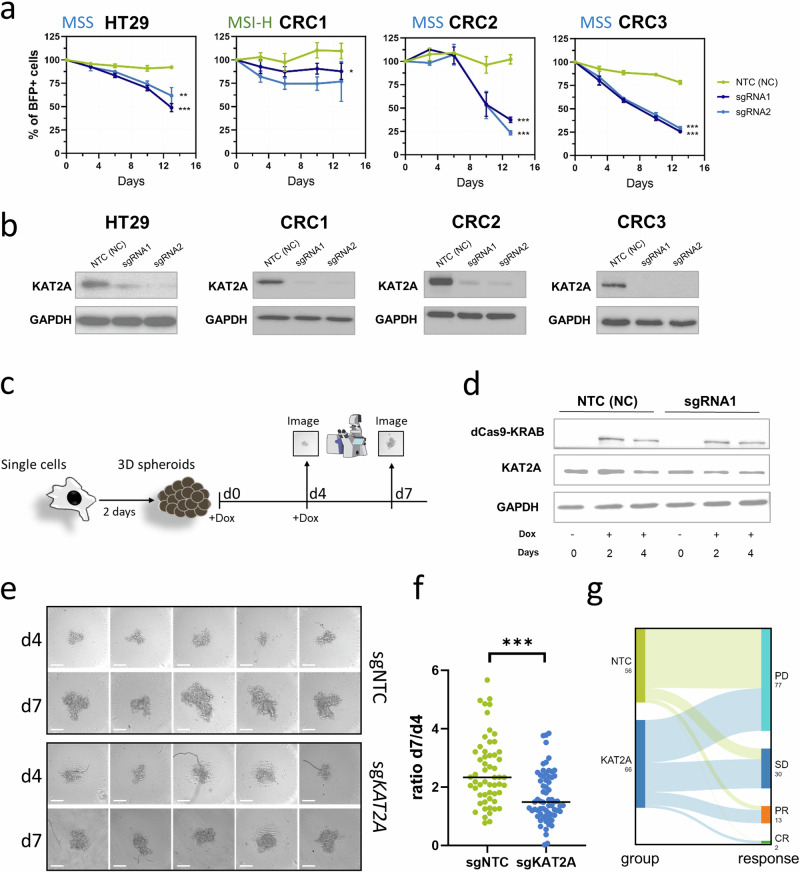
CRISPRi-based knockdown of *KAT2A* reduces CRC tumor progression. **a** Dependency on *KAT2A* was validated using a flow cytometry-based competition assay in the MSS CRC cell line HT29 and in three patient-derived 3D spheroid models (MSI-H CRC1 and MSS CRC2/CRC3) with two constitutive CRISPRi sgRNAs. A non-targeting control (NTC) sgRNA served as a negative control. BFP was used as a marker for sgRNA-expressing cells. *p < 0.05; **p < 0.01; ***p < 0.001, two-tailed Student’s *t*-test. **b** The efficacy of sgRNA-based knockdown was assessed by western blot. **c** Experimental workflow to study the effect of a dox-inducible knockdown of *KAT2A* in already-formed tumor spheroids. Singularized CRC3-dox-dCas9-KRAB cells were cultured in single wells of a 384-well plate until visible 3D structures formed. Subsequently, doxycycline was added to induce the knockdown of *KAT2A*. Spheroid size was assessed by microscopy at four (d4) and seven days (d7) post-doxycycline supplementation. **d** Efficacy of doxycycline-induced dCas9-KRAB induction and the associated reduction of KAT2A was assessed by western blot. **e** Representative images of d4 and d7 spheroids from the NTC-transduced CRC3 spheroids (top) and the *KAT2A* sgRNA-transduced CRC3 spheroids (bottom). Scale bar = 100 µm. **f** Summary of the calculated size ratios (d7/d4) in the NTC control and *KAT2A* knockdown from all included single spheroids. *** *p* < 0.001, two-tailed Mann–Whitney *U* test. **g** Sankey plot summarizes the response of each spheroid from the NTC and sgRNA *KAT2A* groups. Individual spheroids were categorized into four response groups: progressive disease (PD), if the size at day 7 (d7) was ≥1.5-fold larger compared to day 4 (d4); stable disease (SD), if d7 was between 1- and 1.5-fold larger compared to d4; partial remission (PR), if d7 was smaller than d4; and complete remission (CR), if the spheroid completely disappeared.

To validate the database findings, we conducted a loss-of-function study using CRISPR interference (CRISPRi)-mediated knockdown. We utilized one microsatellite instability-high (MSI-H) patient-derived 3D CRC model (CRC1) and three MSS models: the *KAT2A*-dependent CRC cell line HT29 and two patient-derived 3D CRC spheroid models, designated as CRC2 and CRC3. In brief, all models were transduced to stably express dCas9-KRAB alongside GFP. Subsequently, two constitutive sgRNAs, along with a BFP reporter gene, were used to target *KAT2A*. A non-targeting control sgRNA (NTC) was included as a negative control. The relative growth of cells with KAT2A knockdown (BFP+) compared to non-transduced cells (BFP−) was monitored using a flow cytometry-based competition assay. In all MSS models, the *KAT2A* knockdown resulted in significantly reduced growth, as indicated by a decrease in the proportion of BFP+ cells for both sgRNAs. In contrast, the MSI-H model CRC1 did not exhibit this effect (Fig. 3a). On-target editing was confirmed at the protein level by western blot (Fig. 3b) and at the transcript level by qPCR (Supplementary Fig. S2a), demonstrating high knockdown efficiency of the CRISPRi system in all models.

To evaluate the therapeutic potential of KAT2A inhibition, we sought to induce the KAT2A knockdown after the formation of 3D spheroids, mimicking the clinical scenario in which most CRCs are diagnosed at advanced stage when complex 3D tumor structures are already established [35]. For this, we transduced the patient-derived CRC3 model with a doxycycline-inducible dCas9-KRAB and a sgRNA targeting KAT2A, or NTC. Following selection to obtain a pure dox-dCas9-KRAB + /sgRNA+ cell population, we seeded single cells into individual wells of a 384-well plate and cultured them until visible 3D spheroids were formed. Subsequently, doxycycline was added to induce the dCas9-KRAB fusion protein expression. Spheroid sizes were monitored on day 4 (d4) and 7 (d7) after doxycycline supplementation (Fig. 3c). This strategy led to an induced dCas9-KRAB expression and resulted in a mild reduction of KAT2A protein level (Fig. 3d). Imaging and size analysis revealed significantly reduced spheroid growth in the KAT2A-targeting sgRNA group compared to the NTC group, although complete tumor progression arrest was not achieved (Fig. 3e, f).

To further evaluate individual spheroids, we categorized them into four groups: progressive disease (PD) if their size at d7 was ≥1.5-fold larger compared to d4; stable disease (SD) if d7 was between 1- and 1.5-fold larger compared to d4; partial remission (PR) if d7 was smaller than d4; and complete remission (CR) if the spheroid completely disappeared. Notably, in the sgRNA-*KAT2A* group, 34 out of 66 spheroids (52%) exhibited growth arrest, including two spheroids that disappeared entirely (CR). In contrast, only 11 out of 56 spheroids (20%) in the NTC group demonstrated comparable effects (Fig. 3g). These findings underscore the potential of KAT2A inhibition as a therapeutic strategy to impair tumor growth in established CRC spheroids, even when complete KAT2A loss is not achievable.

### CRISPR interference-mediated knockdown of *KAT2A* diminishes proliferation and stemness while promoting expression of differentiation markers

**Fig. 4:**
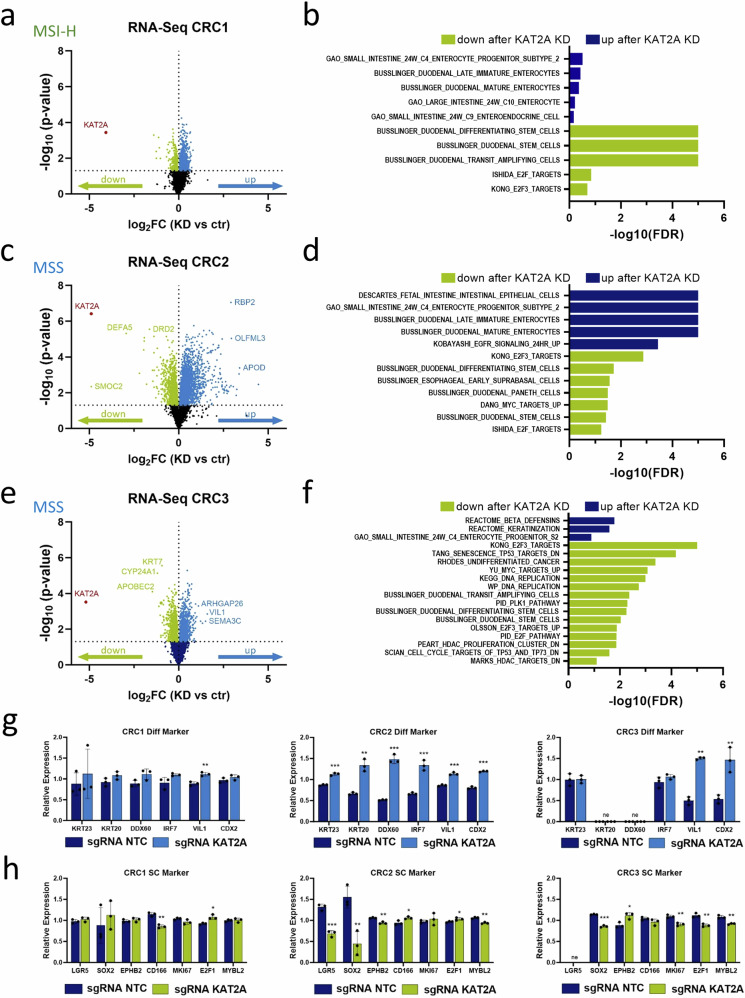
A CRISPRi-based knockdown of *KAT2A* reduces proliferation and stemness, and induces expression of differentiation markers. **a** Quantitative transcriptomics by RNA sequencing are shown as volcano plot and highlight differentially expressed genes between *KAT2A*-knockdown (green) and NTC control (blue) in the MSI-H patient-derived CRC1 model. **b** GSEA results of most significantly upregulated or downregulated signatures in CRC1 cells after *KAT2A* knockdown. **c** Quantitative transcriptomics by RNA sequencing are shown as volcano plot and highlight differentially expressed genes between *KAT2A*-knockdown (green) and NTC control (blue) in the MSS patient-derived CRC2 model. **d** GSEA results of most significantly upregulated or downregulated signatures in CRC2 cells after *KAT2A* knockdown. **e** Quantitative transcriptomics by RNA sequencing are shown as volcano plot and highlight differentially expressed genes between *KAT2A*-knockdown (green) and NTC control (blue) in the MSS patient-derived CRC3 model. **f** GSEA results of most significantly upregulated or downregulated signatures in CRC3 cells after *KAT2A* knockdown. **g** Gene expression overview of differentiation markers *KRT23*, *KRT20, DDX60, IRF7, VIL1* and *CDX2* in all three models. Data were obtained from the RNA-sequencing approach. **h** Gene expression overview of stem cell markers *LGR5*, *SOX2*, *EPHB2*, *CD166*, and proliferation markers *MKI67*, *E2F1* and *MYBL2* in all three models. Data were obtained from the RNA-sequencing approach. *p < 0.05; **p < 0.01; ***p < 0.001, two-tailed Student’s *t*-test.

To further explore the biological mechanisms underlying the observed phenotype and the differences between MSI-H *KAT2A*-indpendent and MSS *KAT2A*-dependent CRCs, we performed RNA sequencing analysis to examine transcriptional changes upon *KAT2A* knockdown in all models (Supplementary Table 3). Differentially expressed genes (DEGs) were identified by comparison with the NTC sgRNA control. As expected, *KAT2A* itself was the most downregulated gene across all models, confirming effective gene silencing (Fig. 4a, c, e and Supplementary Fig. S2b). To categorize the global transcriptional changes into functional pathways, we conducted GSEA, which revealed altered gene signatures and cellular expression profiles associated with *KAT2A* knockdown. Interestingly, the MSI-H model CRC1 exhibited a reduction in intestinal stem cell-related signatures, including those for duodenal stem cells and transit-amplifying cells, but did not show evidence of reduced proliferation or an induction of enterocyte-associated genes (Fig. 4b). In contrast, the MSS models CRC2 (Fig. 4d), CRC3 (Fig. 4f), and HT29 (Supplementary Fig. S2c) displayed an increase in enterocyte progenitor signatures following *KAT2A* knockdown. Moreover, the MSS models showed signs of reduced proliferation, including decreased E2F activity, or activation of apoptosis.

To investigate differences in the effects of *KAT2A* knockdown between MSI-H and MSS models in more detail, we examined the relative expression of individual genes associated with differentiation, stemness, and proliferation. Notably, *KAT2A* knockdown induced the expression of several enterocyte and pan-colon differentiation markers, such as *KRT23*, *KRT20*, *DDX60*, *IRF7, VIL1, and CDX2* exclusively in the MSS models (Fig. 4g and Supplementary Fig. S2d). This pattern was absent in the MSI-H CRC1, aligning with the functional phenotype observed upon *KAT2A* knockdown in this model. Additionally, the MSS models exhibited reduced expression of stem cell regulatory genes, such as *SOX2*, along with downregulation of genes involved in cell cycle progression and proliferation, like *MKI67*, *E2F1*, and *MYBL2* (Fig. 4h and Supplementary Fig. S2e). This downregulation was not observed in the MSI-H model and explains the growth progression observed in the competition assay. In summary, these findings demonstrate that CRISPRi-mediated silencing of *KAT2A* selectively impairs CRC cell growth in MSS models. This effect is likely driven by the downregulation of genes critical for stemness and proliferation, coupled with the induction of genes involved in differentiation.

### Chemical inhibition of KAT2A does not fully mimic the genetic *KAT2A*-knockdown

As the CRISPRi gene knockdown approach provided insights into the consequences of *KAT2A* depletion, we sought to validate these findings by directly inhibiting KAT2A at the protein level. First, we utilized three patient-derived 3D spheroid models (CRC1, CRC2, CRC3) and treated them with increasing concentrations of the two chemical HAT/KAT2A inhibitors, MB-3 [36] and CPTH2 [37], to determine the half-maximal inhibitory concentrations (IC50) for each CRC model. This approach aimed to refine the potential therapeutic implications of targeting KAT2A in CRC using small-molecule inhibitors. Unexpectedly, none of the CRC cultures responded adequately to either MB-3 or CPTH2, even at high concentrations (Supplementary Fig. S3a). The only exception was the MSS CRC3 model, which exhibited reduced viability with CPTH2 at 50 µM, a concentration commonly used for this inhibitor [38]. As a result, we proceeded to include only CRC3 and CPTH2 in further analyses.

To evaluate the effects of CPTH2 treatment in CRC3 on proliferation, stemness, and apoptosis, we performed cell cycle analysis using Hoechst staining, assessed the cell surface abundance of the stem cell marker EPHB2 [39], and determined the fraction of apoptotic cells by Annexin V staining. Notably, CPTH2 induced a G0/G1 cell cycle arrest (Supplementary Fig. S3b, c). Additionally, flow cytometry analysis revealed a strong reduction in EPHB2 abundance in CRC3 cells post-treatment with CPTH2 (Supplementary Fig. S3d). At this concentration, CPTH2 also induced apoptosis (Supplementary Fig. S3e, f).

Collectively, these findings suggest that most 3D CRC models are not sensitive to KAT2A/HAT inhibitors MB-3 and CPTH2. However, inhibition of HATs/KAT2A may have the potential to induce cell cycle arrest, differentiation, and apoptosis.

### *KAT2A* depletion inhibits growth of patient-derived xenografts in vivo

**Fig. 5:**
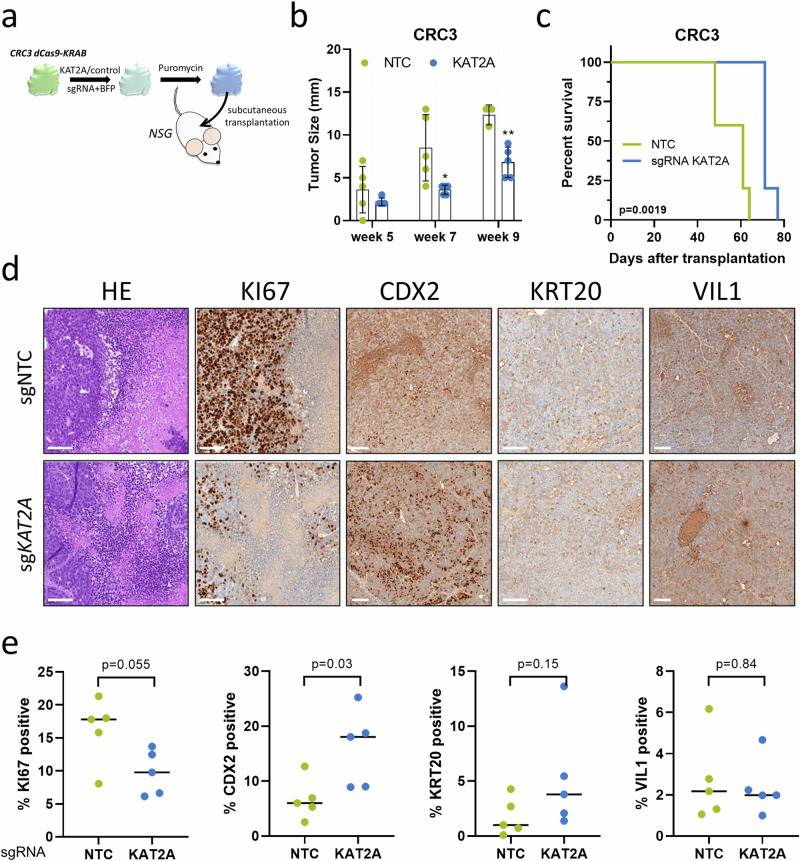
Loss of *KAT2A* markedly decreases tumor formation in vivo. **a** Schematic representation of in vivo validation of KAT2A functional role. CRC3-dCas9-KRAB and GFP expressing patient-derived spheroids, stably expressing either *KAT2A*-targeting or NTC sgRNA, were transplanted subcutaneously into immunodeficient NSG mice to generate xenografts (*n* = 5 per group). **b** Growth rate of tumors measured at defined time points. *p < 0.05; **p < 0.01, two-tailed Student’s *t*-test**. c** Kaplan–Meier survival analysis as mean of the time until the tumors reached the final defined tumor size. *p*-value was determined by Log-rank test. **d** Representative images of hematoxylin-eosin (HE) and immunohistochemistry (IHC) staining for proliferation marker KI67, and differentiation markers CDX2, KRT20, and VIL1 from the final tumors from the NTC and sgRNA-*KAT2A* group. Scale bar = 100 µm. **e** Summary of the calculated percentage of positive cells for each staining from all tumors. *p*-values were determined by two-tailed Mann–Whitney *U* test.

Given the functional role of KAT2A demonstrated in vitro, we sought to validate the effect of KAT2A deficiency on tumor growth in an in vivo setting. This model has the advantage of not being influenced by medium-supplemented growth factors, and the metabolic microenvironment and vascularization are much closer to those of human disease [40]. Thus, we utilized the patient-derived 3D spheroid model CRC3, MSS stable and well-differentiated CRC model with no detectable expression of the stem cell marker *LGR5*, which stably expressed the dCas9-KRAB fusion protein and a GFP reporter. These cells were transduced with either *KAT2A* sgRNA1 or NTC sgRNA along with a BFP reporter, then selected with puromycin for two days to obtain a purified BFP+ population. Subsequently, we subcutaneously injected these cells into immunodeficient non-obese diabetic (NOD)/severe combined immunodeficiency (SCID)/gamma (NSG) mice (Fig. 5a). Tumor growth was frequently monitored, and the mice were sacrificed when the tumors reached an average diameter of 13 mm. Although the time until tumors became visible and palpable did not differ between conditions, the *KAT2A* knockdown group exhibited significantly delayed tumor growth over time compared to the control (Fig. 5b). This led to a significant increase in the overall time until the tumors reached the final defined size (Fig. 5c). After reaching the defined size, the tumors were excised, documented macroscopically, and transferred to 4% paraformaldehyde (PFA) for histological processing. Formalin-fixed, paraffin-embedded (FFPE) tumor slides were then analyzed by immunohistochemical staining to assess tumor morphology and the expression of the proliferation marker KI67 (Supplementary Fig. S4a, b). As expected, xenograft tumors exhibited the typical histology of CRC adenocarcinomas, expressing the proliferation marker KI67 and partially also differentiation markers such as CDX2, particularly following *KAT2A* knockdown. By determining the percentage of positive cells for each marker, we observed a significantly increased percentage of CDX2-positive cells following *KAT2A* knockdown. We also noted a trend toward reduced KI67 expression and higher KRT20 levels in tumors from the *KAT2A* knockdown group compared to control mice (Fig. 5e). Together, these results suggest that *KAT2A* deficiency reduces tumor growth and improves survival in vivo.

### Genes associated with differentiation act as biomarkers indicating *KAT2A* dependency

**Fig. 6:**
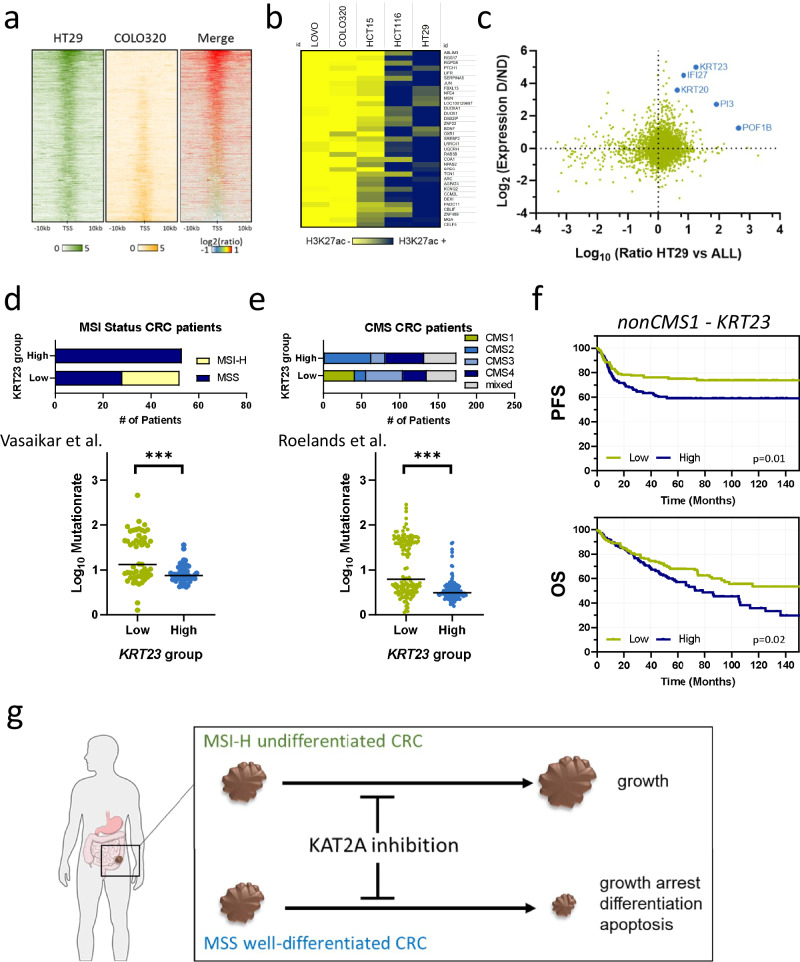
Differentiation-associated genes serve as biomarkers for *KAT2A* dependency. **a** Average line plot of mean H3K27ac read density close to transcriptional start sites (TSS) in *KAT2A* dependent HT29 and *KAT2A* independent COLO320 cells. Dark lines indicate stronger signals, and red plot represents the ratio merge between HT29 versus COLO320 cells. **b** Heatmap of overlapping hit genes with strong H3K27ac signals in *KAT2A* dependent HCT116 and HT29 cells versus *KAT2A* independent LOVO, COLO320, and HCT15 cells. **c** Scatter plot of differentially expressed genes between *KAT2A* dependent versus *KAT2A* independent CRC cell lines (y-axis) and the differentially acetylated gene loci at H3K27 in HT29 versus the *KAT2A* independent CRC cell lines LOVO, COLO320, and HCT15 (x-axis). *KRT23*, *IFI27*, *KRT20*, *PI3*, and *POF1B* are highlighted. **d** CRC patients from the Clinical Proteomic Tumor Analysis Consortium trial (CPTAC-2, Vasaikar et al.) were stratified according to their median *KRT23* expression into two groups (low versus high, *n* = 53 per group). MSI-H is only present in the low-*KRT23* expressing group, and this group associates with a significantly higher mutation count compared to the high-*KRT23* group. ***p < 0.001, two-tailed Mann–Whitney *U* test. **e** CRC patients from the Sidra-LUMC AC-ICAM trial (Roelands et al.) were stratified into two groups based on median *KRT23* expression (low versus high, *n* = 174 per group). *KRT23*-high CRC patients are predominantly classified within the CMS2 and CMS4 subtypes, with no representation in the CMS1 subtype. This classification is reflected in the mutational burden, which is significantly higher in the *KRT23*-low group. *** *p* < 0.001, two-tailed Mann–Whitney *U* test. **f** Non-CMS1 CRC patients from the Sidra-LUMC AC-ICAM trial were divided based on median *KRT23* expression (low versus high, *n* = 153 per group). High *KRT23* expression is associated with shorter progression-free survival (PFS; *p* = 0.01) and shorter overall survival (OS; *p* = 0.02). *p*-values were calculated using the log-rank test. **g** A schematic model illustrating the major findings of this study. We demonstrate that a subset of MSS, more-differentiated CRC subtypes, depends on KAT2A, whereas KAT2A is not essential in MSI-H CRCs.

KAT2A is a HAT that facilitates gene transcription by modifying the epigenetic landscape primarily by acetylating multiple lysine residues on histone 3 (H3), especially lysine 9 (H3K9), but also lysine 27 (H3K27) [9, 41]. Moreover, H3K27 acetylation has been linked primarily to actively transcribed loci [42]. We were therefore interested in determining whether there are differences in H3K27 acetylation between *KAT2A-*independent and *KAT2A-*dependent cell lines. Therefore, we utilized two published Chromatin Immunoprecipitation (ChIP)-Sequencing datasets [23, 24], and included H3K27ac marks in 5 CRC cell lines. Specifically, we compared the acetylation levels within ±10kb window of the transcription start site, represented by H3K27ac enriched regions, between the *KAT2A* dependent HT29 cell line (dependency score = −0.47), the *KAT2A* intermediate dependent HCT116 (dependency score = −0.31), and the three *KAT2A* independent cell lines COLO320 (dependency score = 0.11), LOVO (dependency score = 0.12), and HCT15 (dependency score = 0.02). Interestingly, stronger signal for the majority of genes was observed in the HT29 cell line compared to COLO320 cells, suggesting a more open chromatin architecture in this *KAT2A*-dependent cell line (Fig. 6a). As further validation, we examined H3K27ac profile of genes found to be common hits in HT29 and HCT116 cells. We then compared those profiles to the H3K27ac levels for the same genes in LOVO, COLO320 and HCT115. This analysis demonstrated a clear clustering of *KAT2A-*depended and independent models based solely on the H3K27ac profile of the identified genes, providing further support for the presence of distinct H3K27ac acetylation pattern (Fig. 6b).

Next, we integrated HT29-specific H3K27ac enriched genes with differentially expressed genes between *KAT2A* dependent and independent cell lines identified from transcriptomic data. Strikingly, the previously identified *KAT2A* dependency surrogate marker *KRT23*, in addition to displaying differential expression, exhibited a higher proportion of H3K27ac marks at its respective loci (Fig. 6c).

Lastly, we aimed to determine whether the expression of *KAT2A* dependency biomarker *KRT23* could also serve as surrogate marker for *KAT2A* dependency in CRC patients. To this end, we utilized transcriptomic data from a cohort of 110 CRC patients, generated by the Clinical Proteomic Tumor Analysis Consortium (CPTAC-2) [43] and the Sidra-LUMC AC-ICAM trial [44]. Patients were stratified into two groups—*KRT23* low and *KRT23* high—based on median *KRT23* mRNA expression. In line with our findings in cell lines, *KRT23*-high patients were exclusively MSS and exhibited a lower mutational burden compared to *KRT23*-low patients in the CPTAC-2 dataset (Fig. 6d). In the Sidra-LUMC AC-ICAM trial, the *KRT23*-high group lacked patients from consensus molecular subtype 1 (CMS1), the subtype associated with MSI-H CRC [44]. In contrast, this group consisted primarily of CMS2 and CMS4 CRC subtypes, and generally associated with a lower mutational burden (Fig. 6e). To investigate the potential of *KRT23* expression as a biomarker for patient outcome in MSS CRC patients, we also assessed the survival of both groups. Patients from the CMS1 subgroup were excluded, as this group primarily comprises MSI-H patients that harbor a low *KRT23* expression and shorter survival, which could introduce bias. Notably, higher *KRT23* expression was associated with shorter progression-free survival (PFS) and overall survival (OS) (Fig. 6f). In contrast, a similar approach that included all patients with unknown CMS classification from the public TCGA CRC dataset [26] was unable to predict survival based on *KRT23* expression (Supplementary Fig. S5a–g).

Collectively, our data indicate that KAT2A is a promising therapeutic target for a subset of MSS, more-differentiated CRC patients (Fig. 6g). In addition, based on expression, acetylation, and patient data, *KRT23* might be a promising surrogate biomarker for *KAT2A* dependency in a clinical setting.

## Discussion

While chemotherapy remains a cornerstone in the first line systemic treatment of many cancers, targeted therapies have shown promising results in clinical trials and have initiated the era of precision oncology [45]. In CRC, successful incorporation of immune checkpoint inhibitors and the inhibition of tyrosine kinase receptors EGFR and VEGFR into routine treatment regimens have been achieved [35]. Moreover, other potential therapeutic intervention points have been proposed, such as the HGF/cMET pathway [46], the Notch pathway [47], or the IGF-1R pathway [48]. However, as the long-term survival rates of advanced stage CRC are still poor [49], novel therapeutic targets must be discovered and corresponding biomarkers should be explored.

In this study, we introduce histone acetyltransferase KAT2A as a potential novel vulnerability in distinct CRC subtypes. KAT2A catalyzes the attachment of acetyl groups on several H3 sites, including H3K9, H3K14, and partially H3K27 [9, 41]. These histone modifications are believed to enhance active gene expression, with KAT2A primarily responsible for maintaining, rather than initiating, transcriptional activation [50]. KAT2A, specifically, and histone acetyltransferases (HATs) in general, are counteracted by histone deacetylases (HDACs), and preserving a balance between HATs and HDACs is crucial for normal transcriptional regulation [51]. Notably, CRC often exhibits abnormal epigenetic modifications, such as changes in histone acetylation profiles [52]. Both HAT inhibitors as well as HDAC inhibitors have been suggested as potent therapeutic options in preclinical models and clinical trials for various cancers, including CRC [53]. Furthermore, it has been demonstrated that *KAT2A* expression is elevated in CRC, and the knockdown of *KAT2A* has been shown to reduce the proliferation and migration of CRC cell lines [11, 54].

We demonstrated that *KAT2A* is essential for approximately one-third of all CRC cell lines included in the DepMap dataset [28]. Interestingly, we observed that *KAT2A* expression itself is not a reliable predictor for *KAT2A* dependency. Instead, especially MSS CRCs with differentiation-associated transcriptional profiles, characterized by high expression of enterocyte-specific marker genes and the expression of keratin genes *KRT20* and *KRT23*, depend on *KAT2A*. CRC is characterized by high heterogeneity and was originally classified into four histological grades based on tumor differentiation, with less-differentiated tumors associated with the worst prognosis [55] and higher frequency of MSI-H [56], a clinical feature that we identified as mutually exclusive with *KAT2A* dependency. This aligns with previous data suggesting that MSI-H CRC tumors, which also present with higher mutation rates, are more driven by genetic events [57]. Although there is evidence for increased DNA methylation in MSI-H CRCs [58], the data about histone modification difference between MSS and MSI-H CRCs are rare. It has been reported that the histone modifier NSD2 is less active in MSI-H CRCs compared to MSS CRC [59]. However, recent evidence suggests that histological classification does not necessarily correspond to gene expression profiles in CRCs, and the development of the consensus molecular subtype (CMS) has improved CRC stratification [58]. Moreover, molecularly more-differentiated CRCs can also be linked to adverse prognosis, for instance, if they exhibit gene expression signatures related to enterocyte-like cells [60]. We identified KAT2A as an essential component especially in CRC cells with increased enterocyte gene signatures and MSS background. As the CMS1 is strongly associated with MSI-H status [58], KAT2A might be a potential target only in non-CMS1 CRC patients.

We identified expression of various colon differentiation-associated genes, such as *KRT20* and *KRT23*, as promising surrogate markers for KAT2A-dependency. KRT20 is a marker of differentiated colon cells, and high *KRT20* expression is associated with more differentiated CRC subtypes [61, 62]. In contrast, KRT23 is a differentiation marker in MSS CRCs and has a tumor suppressive function in MSI-H CRC [63]. Moreover, it is an epithelial-specific intermediate filament protein that is associated with epithelial differentiation [64]. One can speculate that more differentiated CRCs, with a generally lower mutation load [56], are more likely to be epigenetically driven. CRCs display remarkable plasticity, and CRC cells with enterocyte-specific gene expression signatures can also serve as cancer stem cells [65]. As a result, these subtypes already exhibit active transcriptional programs of normal colon differentiation, likely because they arise at later stages of colon development [66]. Consequently, re-inducing terminal differentiation, associated with cell-cycle arrest, and subsequent cell death requires fewer additional transcriptional activation steps. We hypothesize that in molecularly more differentiated tumors, reducing the expression of self-renewal programs may be sufficient to induce differentiation. This aligns with previous findings suggesting that two hits, blocking stem cell programs and inducing differentiation, are necessary to reintroduce differentiation in undifferentiated CRCs [67]. Consistent with our findings, the protein arginine methyltransferase (PRMT) type 1 inhibitor MS023 has been shown to re-induce differentiation in CRC and to increase expression of differentiation markers such as CDX2 [68].

Consistent with our data, global histone acetylation levels are elevated in more differentiated CRCs [69]. Our study demonstrated that blocking *KAT2A* genetically led to reduced cell growth, diminished stemness, and induced the expression of differentiation-associated genes. Interestingly, we observed that the knockdown of *KAT2A* led to a significant decrease in the expression of various genes, including those responsible for stem cell self-renewal and proliferation in CRC [39]. This effect is likely attributed to KAT2A’s role as a transcriptional activator, where the immediate consequences of its knockdown primarily involve silencing gene expression. In agreement with this, we also observed an activation of HDAC target genes in HT29 cells upon KAT2A inhibition, and we hypothesize that KAT2A as a HAT competes with various HDACs for histone sites. Additionally, we also observed an upregulation of differentiation-associated genes upon KAT2A inhibition. We hypothesize that these are downstream effects, which are not directly mediated by altered histone acetylation. Generally, changes in cellular phenotypes may be delayed and only become apparent at later stages following *KAT2A* depletion. Similar effects on global transcriptional changes have been reported for the inhibition of other HATs, as for targeting p300/CBP with the small molecule inhibitor A-485 in lymphoid malignancies [70].

Taken together, the dependency on *KAT2A* in CRC is closely associated with features of enterocytic differentiation and MS status. Inhibiting KAT2A in well-differentiated CRC enhances maturation and induces cell growth arrest. Further investigation is needed to determine whether KAT2A could serve as a clinically applicable target in patients.

## Supplementary information

Supplementary Figures

Supplementary Table 1

Supplementary Table 2

Supplementary Table 3

Supplementary Material File

## Data Availability

RNA sequencing data have been deposited in the Gene Expression Omnibus (GEO) Repository with the Accession Number GSE268709 and GSE287741 and are publicly available. Processed dependency and expression data files are listed in the Supplementary Table section. The links to public datasets analyzed in this study are reported in the manuscript.
