# Supplementary material for: The histone modifier KAT2A presents a selective target in a subset of well-differentiated microsatellite-stable colorectal cancers
Source: Cell Death Differ. 2025 Mar 27;32(7):1259–72. doi: 10.1038/s41418-025-01479-7 (PMC12284170; doi:10.1038/s41418-025-01479-7)
Supplement: Supplementary file 1 — Supplementary Figures [file 41418_2025_1479_MOESM1_ESM.pdf]

SUPPLEMENTARY FIGURE 1

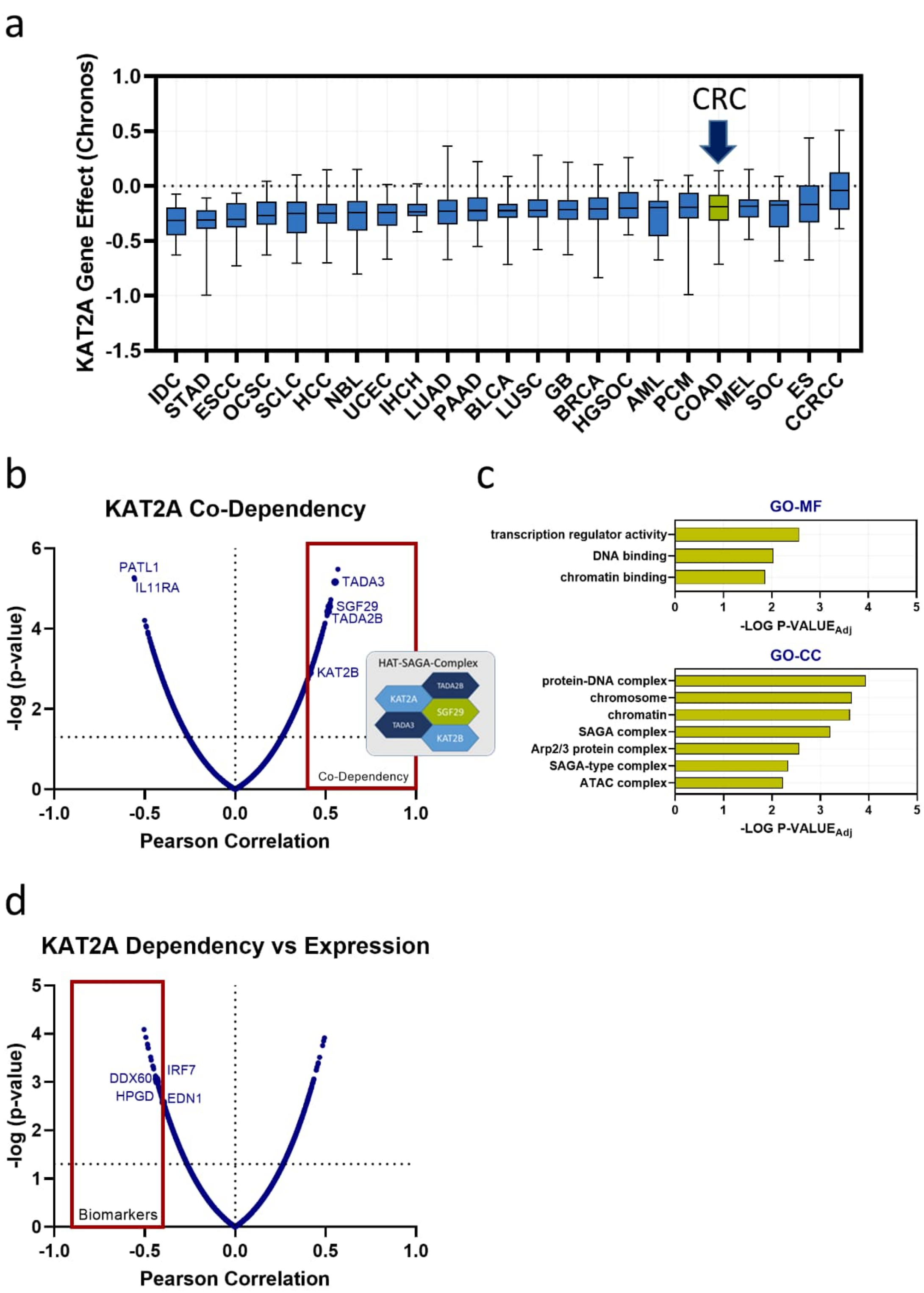

Supplementary Figure S1. *KAT2A* dependency correlates with dependency of SAGA-complex members and expression of differentiation markers.

a Overview of the *KAT2A* dependency score categorized into entities in pan-cancer cell lines (n=1078) from the DepMap database. Entities were ranked according to the median dependency score for

KAT2A, starting with the strongest dependency. IDC = Intraductal Papillary Neoplasm of the Bile Duct; STAD = Stomach Adenocarcinoma; ESCC = Esophageal Squamous Cell Carcinoma; OCSC = Oral Cavity Squamous Cell Carcinoma; SCLC = Small Cell Lung Cancer; HCC = Hepatocellular Carcinoma; NBL = Neuroblastoma; UCEC = Uterine Corpus Endometrial Carcinoma; IHCH = Intrahepatic Cholangiocarcinoma; LUAD = Lung Adenocarcinoma; PAAD = Pancreatic Adenocarcinoma; BLCA = Bladder Urothelial Carcinoma; LUSC = Lung Squamous Cell Carcinoma; GB = Glioblastoma; BRCA = Breast Invasive Carcinoma; HGSOC = High-Grade Serous Ovarian Cancer; AML = Acute Myeloid Leukemia; PCM = Plasma Cell Myeloma; COAD = Colon Adenocarcinoma; MEL = Melanoma; SOC = Serous Ovarian Cancer; ES = Ewing Sarcoma; CCRCC = Clear Cell Renal Cell Carcinoma. Colorectal Cancer (CRC) is highlighted by a blue arrow. b Correlation between *KAT2A* dependency and other gene dependencies. Within the top co-dependent genes are *TADA3*, *TADA2A*, *SGF29*, and *KAT2B*, all members of the Spt-Ada-Gcn5 acetyltransferase (SAGA)-coactivator complex. c G-profiler analysis of the common gene ontology (GO) terms for top co-dependent (r>0.4) genes. Especially transcriptional regulators, DNA-binding and members of the SAGA-complex show co-dependencies with *KAT2A* in CRC. d Correlation between *KAT2A* dependency and mRNA expression of all expressed genes. Indicated are genes from the enterocyte-specific gene signature.

SUPPLEMENTARY FIGURE 2

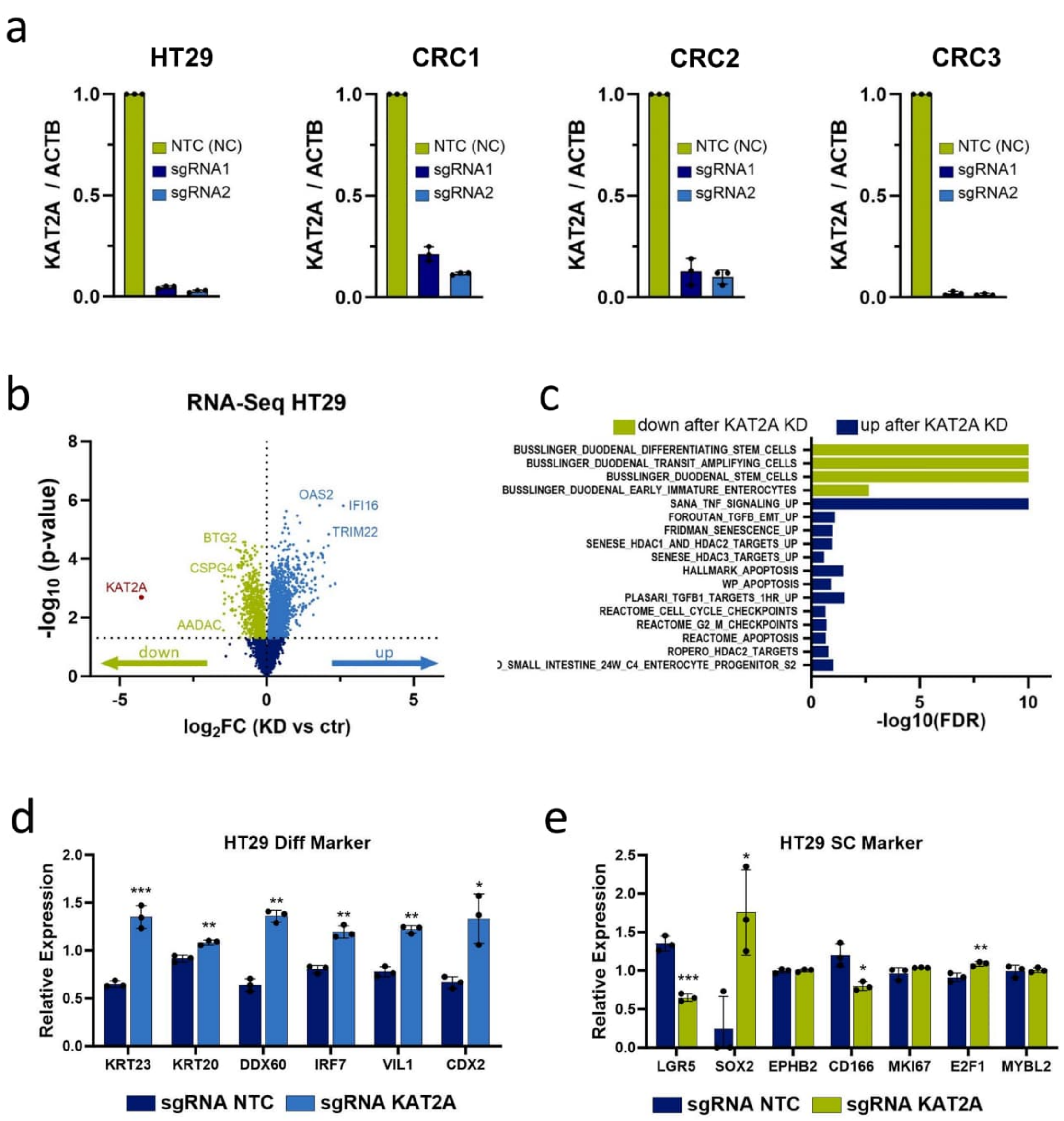

Supplementary Figure S2. CRISPR interference-mediated knockdown of *KAT2A* diminishes proliferation and stemness while promoting expression of differentiation markers in HT29 cell line. a Efficacy of sgRNA-based knockdown in HT29, and the patient-derived 3D models CRC1, CRC2, and CRC3 was assessed by qPCR for *KAT2A* expression. b Quantitative transcriptomics by RNA sequencing are shown as volcano plot and highlight differentially expressed genes between *KAT2A*-knockdown (green) and NTC control (blue) in HT29 cells. c GSEA results of most significantly upregulated or downregulated signatures in HT29 cells after *KAT2A* knockdown. d Gene expression overview of differentiation markers *KRT23*, *KRT20*, *DDX60*, *IRF7*, *VIL1* and *CDX2* in HT29 cells. Data were obtained from the RNA-sequencing approach. e Gene expression overview of stem cell markers *LGR5*, *SOX2*, *EPHB2*, *CD166*, and proliferation markers *MKI67*, *E2F1* and *MYBL2* in HT29 cells. Data were obtained from the RNA-sequencing approach. * $p<0.05$; ** $p<0.01$; *** $p<0.001$, two-tailed Student's t-test.

SUPPLEMENTARY FIGURE 3

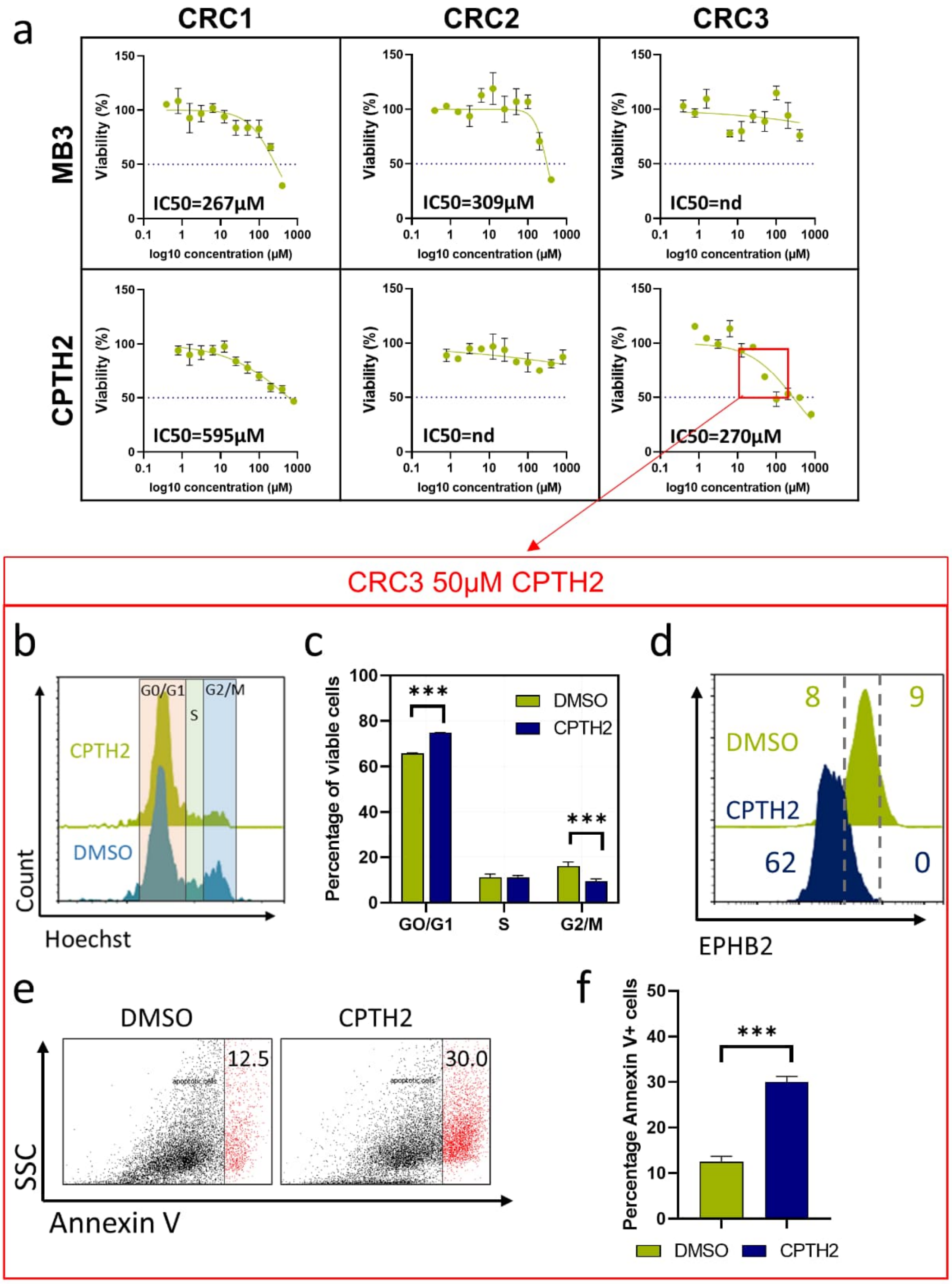

Supplementary Figure S3. Chemical HAT inhibition with 50µM CPTH2 reduces cell growth and induces apoptosis in CRC3.

a IC50 determination for HAT inhibitors MB-3 and CPTH2 in CRC1, CRC2, and CRC3 using ATPlite assay. b Representative histogram for cell cycle analysis of CRC3 treated with 50µM of CPTH2, and c summary of three independent repetitions for treatment with DMSO or 50µM CPTH2. Shown are the percentages of cells in G0/G1, S, and G2/M phase of the cell cycle. d Abundance of stem cell marker

EPHB2 in CRC3 four days after treatment with DMSO or 50µM CPTH2. Fluorescence intensity was determined by flow cytometry and is illustrated as representative example of three independent biological replicates. e Representative dot plot for Annexin V positivity of CRC3 treated with DMSO or 50µM CPTH2, and f summary of three independent replicates. ** $p<0.01$; *** $p<0.001$, two-tailed Student's t-test.

SUPPLEMENTARY FIGURE 4

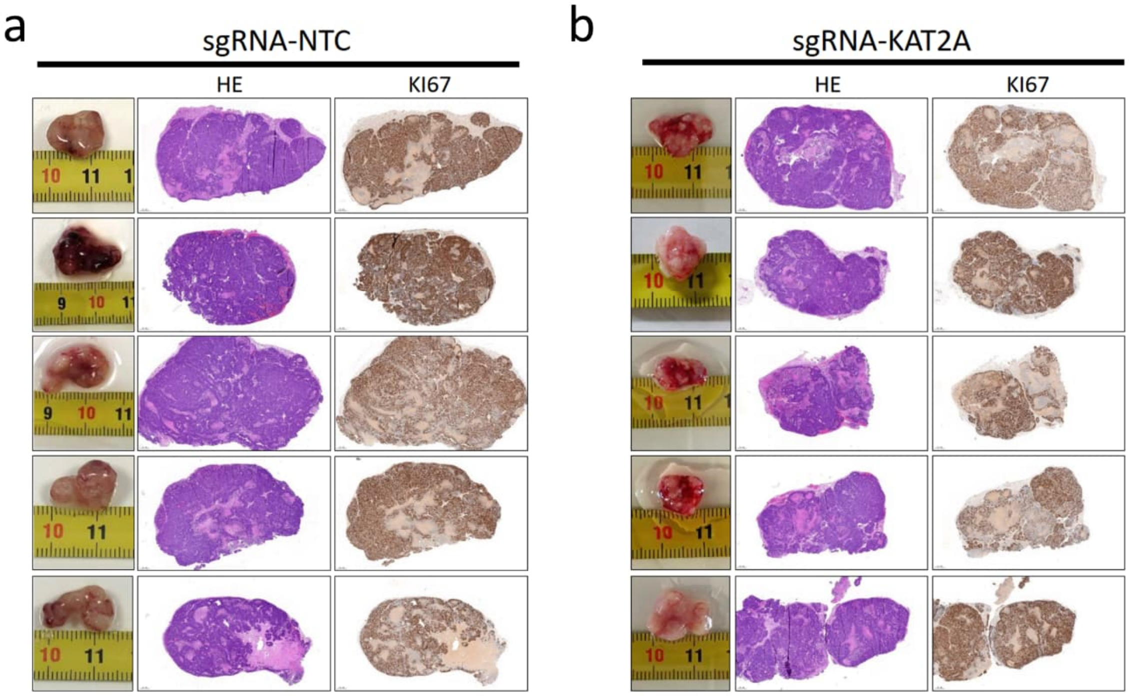

Supplementary Figure S4. CRC3-induced tumors *in vivo* represent the morphology of human CRC tumors.

a–b Macroscopic representation of final tumors and immunohistochemistry staining of the excised tumors for NTC (a) and sgRNA-*KAT2A* (b) group after the final tumor size was reached illustrating the typical CRC adenocarcinoma morphology. The images illustrate the whole tumor signals for HE and KI67 staining.

SUPPLEMENTARY FIGURE 5

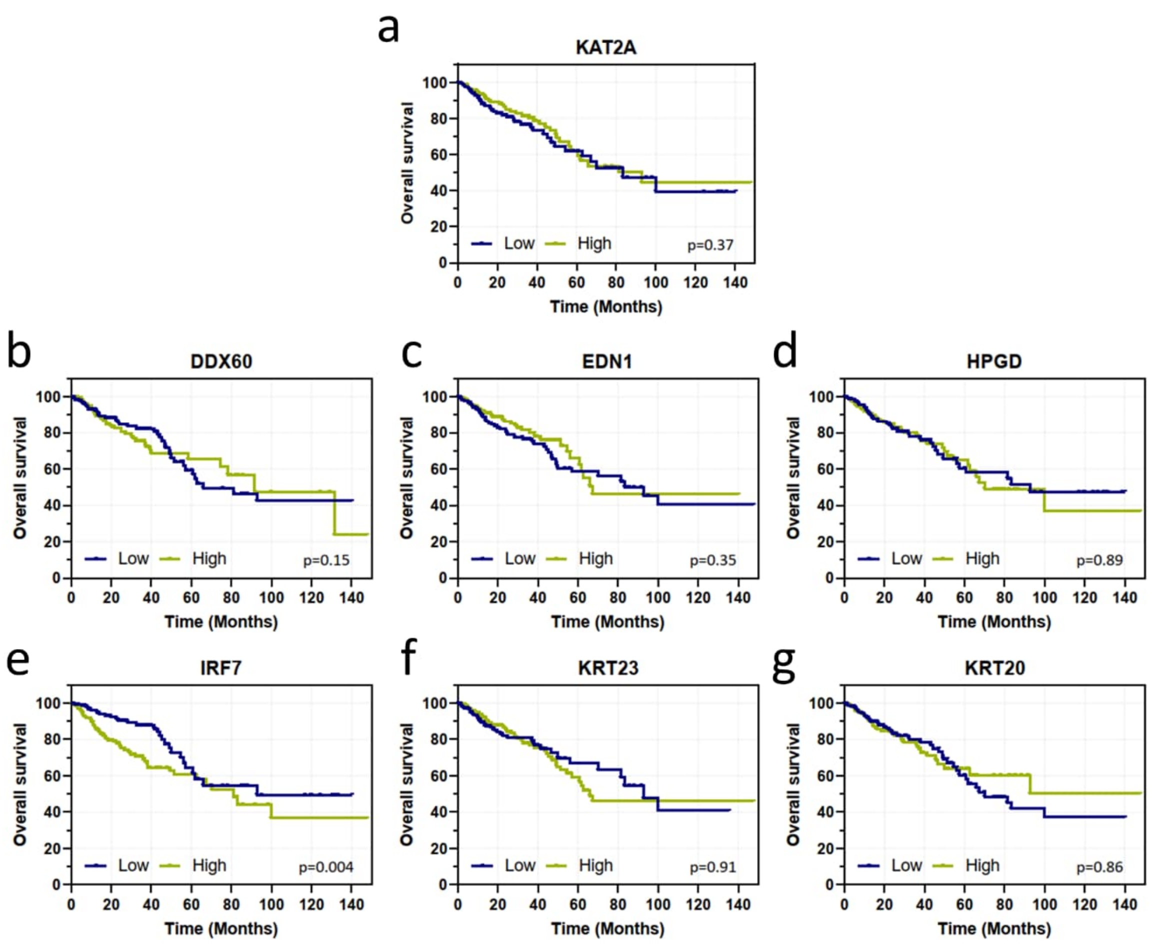

Supplementary Figure S5. Impact of *KAT2A* expression and expression of *KAT2A* dependency surrogate markers on overall survival of CRC patients.

a-g CRC patients (TCGA-COAD, n= 373) were stratified according to the median expression of a *KAT2A*, b *DDX60*, c *EDN1*, d *HPGD*, e *IRF7*, f *KRT23*, and g *KRT20*, and overall survival was compared.
