# Supplementary figures and images for: The histone modifier KAT2A presents a selective target in a subset of well-differentiated microsatellite-stable colorectal cancers
Source: Cell Death Differ. 2025 Mar 27;32(7):1259–72. doi: 10.1038/s41418-025-01479-7 (PMC12284170; doi:10.1038/s41418-025-01479-7)

# Uncropped original western blots

Figure 3b

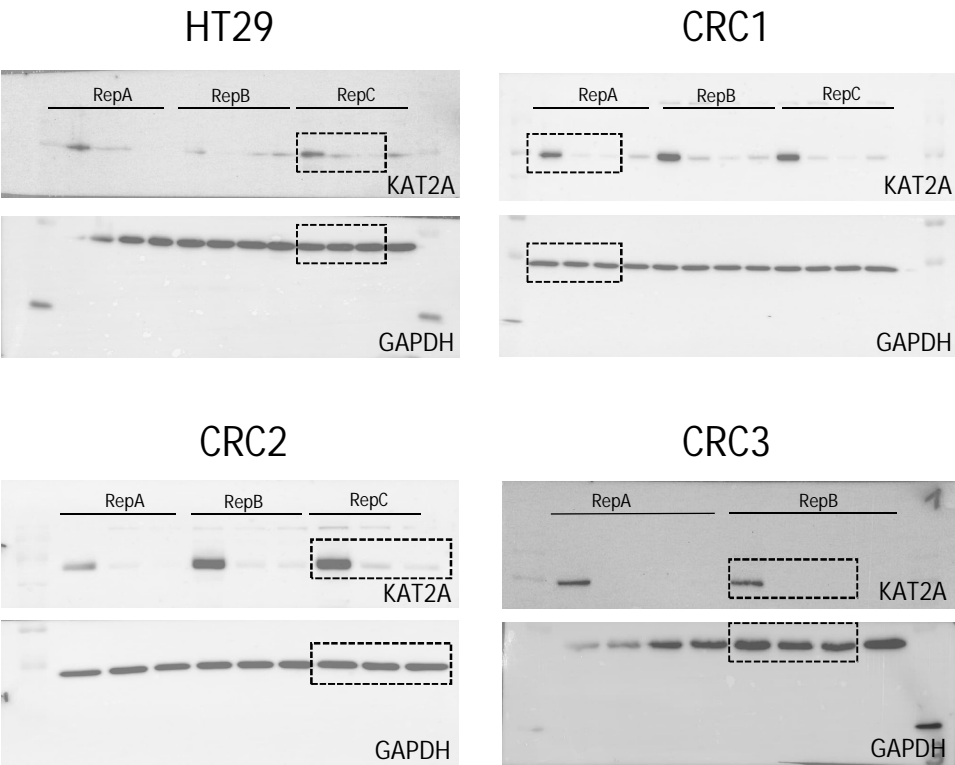

Figure 3d

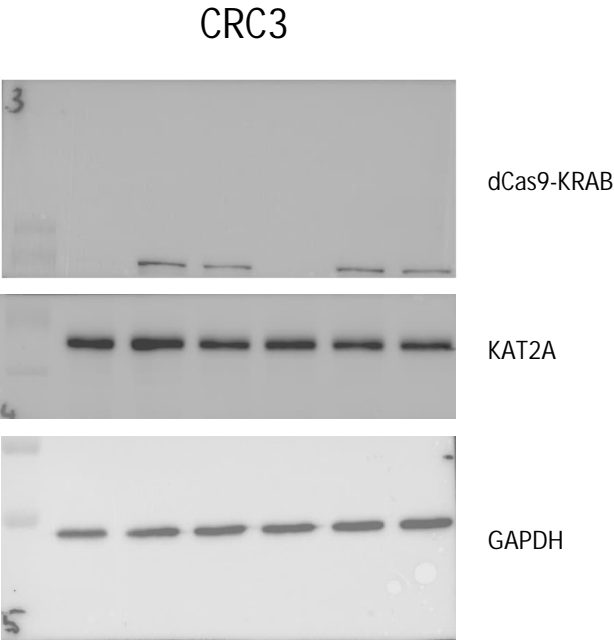

Supplement: Supplementary file 5 — Supplementary Material File [file 41418_2025_1479_MOESM5_ESM.pdf]
